# Evaluation of a pulsed xenon ultraviolet disinfection system to decrease bacterial contamination in operating rooms

**DOI:** 10.1186/s12879-017-2792-z

**Published:** 2017-10-10

**Authors:** Lynn El Haddad, Shashank S. Ghantoji, Mark Stibich, Jason B. Fleming, Cindy Segal, Kathy M. Ware, Roy F. Chemaly

**Affiliations:** 1 Department of Infectious Diseases, Infection Control and Employee Health, The University of Texas MD Anderson Cancer Center, 1515 Holcombe Boulevard, Unit 1460, Houston, TX 77030-4095 USA; 2 Xenex Disinfection Services, San Antonio, TX USA; 3 Department of Gastrointestinal Oncology, H. Lee Moffitt Cancer Center & Research Institute, Tampa, FL USA; 4 Department of Surgical Oncology, Division of Surgery, The University of Texas MD Anderson Cancer Center, Houston, TX USA

**Keywords:** Operating rooms, Environment cleanliness, Pulsed xenon ultraviolet, Between-cases mimicking

## Abstract

**Background:**

Environmental cleanliness is one of the contributing factors for surgical site infections in the operating rooms (ORs). To decrease environmental contamination, pulsed xenon ultraviolet (PX-UV), an easy and safe no-touch disinfection system, is employed in several hospital environments. The positive effect of this technology on environmental decontamination has been observed in patient rooms and ORs during the end-of-day cleaning but so far, no study explored its feasibility between surgical cases in the OR.

**Methods:**

In this study, 5 high-touch surfaces in 30 ORs were sampled after manual cleaning and after PX-UV intervention mimicking between-case cleaning to avoid the disruption of the ORs’ normal flow. The efficacy of a 1-min, 2-min, and 8-min cycle were tested by measuring the surfaces’ contaminants by quantitative cultures using Tryptic Soy Agar contact plates.

**Results:**

We showed that combining standard between-case manual cleaning of surfaces with a 2-min cycle of disinfection using a portable xenon pulsed ultraviolet light germicidal device eliminated at least 70% more bacterial load after manual cleaning.

**Conclusions:**

This study showed the proof of efficacy of a 2-min cycle of PX-UV in ORs in eliminating bacterial contaminants. This method will allow a short time for room turnover and a potential reduction of pathogen transmission to patients and possibly surgical site infections.

## Background

About 400,000 surgical site infections (SSIs) are documented annually in the United States, with associated costs of around $21,000 per case [1, 2]. Prevalent organisms associated with SSIs, such as *Staphylococcus aureus*, *Enterococcus* species, *Klebsiella spp.*, *Pseudomonas aeruginosa,* and *Escherichia coli,* can persist on surfaces from 1.5 h to more than 30 months [3].

Standard manual cleaning alone is not sufficient to eliminate these pathogens; only around 47% of surfaces are appropriately disinfected during between-case and end-of-day terminal manual cleaning [4]. Implementation of efficient environmental disinfection methods as a supplement to manual cleaning may aid in reducing the risk of wound contamination and subsequent infection, thus eliminating the possible transmission of pathogens to patients [5, 6].

The portable ultraviolet light germicidal device employing pulsed xenon lamps (PX-UV) has been shown to be a safe, easy-to-operate, and effective system in decreasing the number of pathogens [7]. PX-UV uses a xenon flash lamp to generate broad-spectrum, high-intensity ultraviolet light to deactivate and kill bacteria, spores, and viruses on high-touch surfaces in 5 min or less [7]. Two studies have shown that the use of PX-UV in addition to standard end-of-day manual cleaning helped reduce bacterial contamination levels on surfaces in the operating rooms (ORs) by 62% and 81% [8, 9].

Furthermore, it was shown that contamination in the OR increases with sequential cases, leading to a more contaminated environment for each subsequent patient during operative hours [9]. Hence, rapid and effective between-case cleaning could reduce environmental contamination, protecting subsequent patients during the same day of operation. While improved patient outcomes have been observed after PX-UV during nightly terminal cleaning practices [8, 9], no data are available on the impact of this technology when applied between surgical cases.

In this study, we aimed to determine the sufficient time required by the PX-UV device to reach environmental cleanliness.

## Methods

This environmental sampling study was conducted at The University of Texas MD Anderson Cancer Center. The sampling occurred between the last end-of-case cleaning and the nightly standard terminal cleaning practices. Cleaning efficacy was assessed after 1, 2, and 8 min of PX-UV cycles using a PX-UV device (Xenex Disinfection Services). These cycle times were chosen based on proof-of-concept experiments conducted in the laboratory setting (data not shown). For each OR, high-touch surfaces were sampled at two distinct time points: after standard end-of-case cleaning and after PX-UV disinfection.

**Fig. 1:**
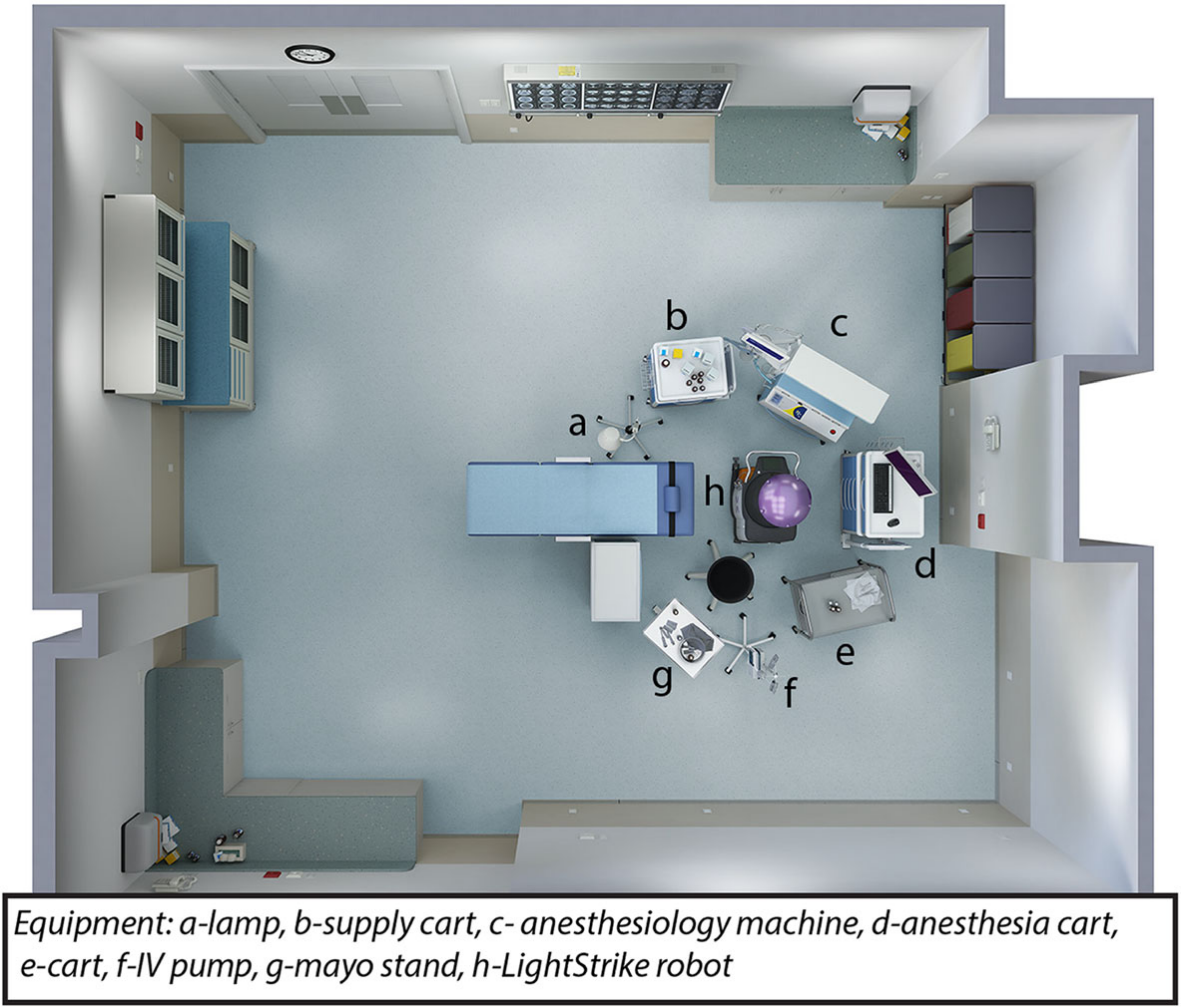
Schematic design of an operating room showing the accurate position of the PX-UV device (purple) to ensure direct line of sight of the UV light to the high-touch surfaces (identified as **a** to **h**)

At the conclusion of surgical cases each day, the room was cleaned by OR staff according to standard end-of-case protocols (manual cleaning with ready-to-use germicidal wipes or diluted solution). Following this cleaning by not more than 1.5 h, samples from 5 high-touch surfaces (computer monitor, electrocautery unit, anesthesia cart, chair, and bed table controls) were collected for quantitative culturing using Tryptic Soy Agar contact plates. For non-flat surfaces, the plates were rolled so that their entire area came in contact with the high-touch surface. The ORs were then disinfected with a PX-UV device for 1, 2, or 8 min (10 rooms for each cycle time) at the head of the table, ensuring direct line of sight for the UV light for high-touch surfaces (Fig. 1). Following PX-UV disinfection, the same 5 high-touch surfaces were sampled at sites adjacent to the first sites using Tryptic Soy Agar contact plates. After 48-h incubation at 37 °C of the plates, colony counts were recorded. We sampled 30 ORs, generating 150 samples before PX-UV use and 150 samples after PX-UV. Table 1 gives a detailed description of all cases in each sampled OR (Table 1).

**Table 1:** Description of the case flow and case types of the 30 sampled operating rooms

OR	PX-UV cycle time (minutes)	Mean c.f.u. before PX-UV	Mean c.f.u. after PX-UV	Total number of cases during the day	Consecutive case type	Last case type of the day before PX-UV
1	1	0.6	0.8	3	Laparotomy; Incision and Drainage; Mastectomy	Mastectomy
2	1	4.2	1.4 ↓	2	Wide Local Excision; Neck Dissection	Neck Dissection
3	1	1.2	1.6	3	Laparoscopic Nephrectomy; Laparoscopic Adrenalectomy	Partial Nephrectomy
4	1	2.3	1.4 ↓	3	Mastectomy, Wide Local Excision	Excision of lesion on back
5	1	3.2	1.4 ↓	1	Laminectomy with stabilization	Laminectomy with stabilization
6	1	2.8	0.4 ↓	2	Nephrectomy	Diagnostic Laparostomy
7	1	6.2	2.2 ↓	2	Thyroid with Neck Dissection	Closure of enterostomy
8	1	2.2	2.6	2	Parotidectomy	Examination under Anaesthesia with biopsies
9	1	4.2	3.6 ↓	1	Removal of tibial nail hardware and complex closure	Removal of tibial nail hardware & complex closure
10	1	1.0	1.6	3	Insertion of Port-A-Cath	Insertion of Port-A-Cath
11	2	0.0	0.0 ↓	1	Partial Excision of genitalia with reconstruction	Partial Excision of genitalia with reconstruction
12	2	1.0	1.2	2	closure of enterostomy	Partial colectomy
13	2	1.8	2.2	1	Laparoscopic Gastrectomy	Laparoscopic Gastrectomy
14	2	1.4	0.6 ↓	2	Tandem and Ovoid insertion	Tandem and Ovoid insertion
15	2	1.0	0.6 ↓	1	Partial Lobectomy Liver and Hysterectomy	Partial Lobectomy Liver and Hysterectomy
16	2	1.4	0.8 ↓	2	Diagnostic Lap with Central Venous Catheter insertion	Diagnostic Laparotomy with CVC insertion
17	2	2.2	0.4 ↓	2	Experimental Laparoscopy with bowel anastomosis	Excision of groin lymph node
18	2	2.6	1.6 ↓	1	Proctectomy, Hysterectomy and reconstruction	Proctectomy, Hysterectomy and reconstruction
19	2	11.8	0.8 ↓	2	Lap Hysterectomy	Lap Hysterectomy
20	2	1.4	0.4 ↓	3	Insertion of central line	Thoracoscopy with segmental lung resection
21	8	1.2	0.6 ↓	1	Craniotomy	Craniotomy
22	8	3.2	1.0 ↓	2	Laparoscopic liver resection	Closure of enterostomy and Laparoscopic liver
23	8	3.2	0.8 ↓	2	Port-A-Cath insertion	Diagnostic Laparotomy with liver biopsies
24	8	4.0	0.6 ↓	2	Mastectomy with reconstruction	Segmental Mastectomy
25	8	10.8	1.6 ↓	1	Mastectomy with reconstruction	Mastectomy with reconstruction
26	8	1.4	0.0 ↓	3	Diagnostic Laparostomy with Hysterectomy; Incision and Drainage cyst	Cystoscopy with biopsies
27	8	0.8	0.2 ↓	2	Laparoscopic Salpingo-Oophorectomy	Diagnostic Laparotomy with biopsies
28	8	2.2	0.6 ↓	2	Thyroidectomy	Mastectomy
29	8	7.8	2.6 ↓	1	Partial colectomy with nephrectomy	Partial colectomy with nephrectomy
30	8	7.6	0.5 ↓	1	Thoracotomy with Lobectomy and Pulmonary Arterial reconstruction	Thoracotomy with Lobectomy and Pulmonary Arterial reconstruction

*Abbreviations: OR, Operating Room; PX-UV, pulsed xenon ultraviolet; c.f.u., Colony-forming units. The symbol “*↓” *indicates a decrease in c.f.u. after PX-UV*

The pre-PX-UV samples were combined for analysis to remove any variance issue. Means, medians, and ranges of colony counts were recorded at each sampling period for statistical analysis. As the data were nonparametric, a Wilcoxon rank sum test was used to examine the differences between groups.

## Results

A total of 147 pre-PX-UV samples and 148 post-PX-UV samples measuring bacterial load obtained for the 5 high-touch surfaces were included in the analysis. Five plates (3 in the pre-PX-UV group and 2 in post-PX-UV groups) were discarded from the analysis as outliers because of counts that were too numerous to count (TNTC) and attributed to lab error, such as a dislodged cover plate. If included in the analysis, the outliers would have had undue leverage on the data for the intervention group that had no outliers (the 1-min group), and therefore the removal of the outliers was deemed conservative.

Table 2 depicts the changes in the colony-forming units (c.f.u.) between pre- and post-PX-UV use at different cycle times. A 1-min cycle of PX-UV did not generate a significant reduction in the level of contamination on the high-touch surfaces (*P* = 0.594). However, 2- and 8-min cycles showed significant reduction in the level of environmental contamination by decreasing the mean colony counts by 72.5% (*P* = 0.0328) and 73.1% (*P* = 0.0075), respectively (Table 2). A 2-min PX-UV cycle was as effective in eliminating an equal load of bacterial contamination when compared to an 8-min cycle.

**Table 2:** Efficacy of 1-, 2-, and 8-min PX-UV disinfection cycle times in reducing operating room contamination

Timing of sampling	Samples taken (*n*)	Colony count (c.f.u.)				Reduction^a^ (%)	*P*-value
		Mean	Min	Max	IQR		
Pre PX-UV (all cycles combined)	147	3.19	0	55	3	–	–
Post 1-min PX-UV	50	1.70	0	14	2	46.7	0.5940
Post 2-min PX-UV	49	0.88	0	9	1	72.5	0.0328
Post 8-min PX-UV	49	0.86	0	7	1	73.1	0.0075

^a^Reduction of mean colony count after PX-UV in comparison with pre-PX-UV mean colony count. PX-UV, pulsed xenon ultraviolet; IQR, Interquartile range; c.f.u., Colony-forming units; min, minimum; max, maximum

## Discussion

We found PX-UV disinfection effective in reducing colony counts when performed after standard cleaning. The 2- and 8-min PX-UV cycles produced equivalent and significant reduction of level of contamination when compared to standard OR cleaning alone and were more effective than the 1-min PX-UV cycle. We conclude that a 2-min cycle optimizes efficacy and efficiency.

A recent meta-analysis of financial impact on the United States healthcare system showed that SSIs contribute to 33.7% of the overall annual cost ($9.8 billion) of healthcare-associated infections [2]. By implementing this SSI prevention approach in the OR setting, contamination in the OR could be controlled during sequential cases, leading to a decontaminated environment for subsequent patients and may have positive impact on the rate of SSIs and associated costs.

PX-UV has been successfully used to reduce or eliminate pathogens such as vancomycin-resistant enterococci, Methicillin-resistant *Staphylococcus aureus* (MRSA), as well as *Clostridium difficile* on high-touch surfaces in patient rooms [10, 11]. In fact, PX-UV combined with quaternary ammonium removed 95% of *C. difficile* spores compared to a 70% of spores reduction when disinfecting patient rooms with bleach [10]. Moreover, PX-UV was 16 times more effective than manual cleaning in eliminating MRSA [12] and 100% effective against VRE [11]. The efficacy of this method has also been confirmed against fungi, *Bacillus anthracis*, and viruses such as Ebola virus [13]. In addition, PX-UV does not damage materials in hospital settings and is not transmitted through glass windows [10].

Another method for decontaminating OR rooms between cases is the use of improved hydrogen peroxide products (IHP) such as Activated Hydrogen Peroxide (Clorox Healthcare). Even though this disinfectant is effective in reducing the contamination level to around 84% of the baseline, it presents a major limitation, i.e., manual cleaning for about 2 to 4.5 h [14]. Manual cleaning is not predictable nor optimal being dependent upon the education of the cleaning personnel and the nurses [15, 16]. In fact, when cleaning, tools such as buckets, mop heads, and wipes can rapidly become contaminated and potentially transfer pathogens to other cleaned surfaces [7]. Also, the continual and recurrent use of the same chemical disinfectant can lead to the emergence of resistant microorganisms [7]. Moreover, the time spent on manually cleaning constitutes an important drawback in ORs where rapid bed turnaround time is crucial and entails operational costs for training specialized personnel. Finally, IHP costs around $175 per room, whereas the PX-UV device costs approximately $3 per room to operate, excluding labor costs in both cases [10].

The present study was limited to 5 high-touch surfaces. Other high-touch surfaces such as floors, light switches, cabinet handles, and doorknobs could be added to future studies. Additional limitations are the somewhat small sample size used in this study and the lack of bacterial identification to the species level by our use of TSA sampling plates, which are limited in detection to aerobic bacteria only. Moreover, the impact of PX-UV use between cases on SSIs and identification of bacteria at the species level on ORs surfaces still need to be determined in future studies. Finally, an operational study that investigate the impact of the between-case use of PX-UV on OR case flow would be necessary.

## Conclusions

In summary, our results suggest that supplementing standard cleaning procedures using a portable no-touch PX-UV system could be done routinely and rapidly between cases in the OR. A cycle of 2 min was sufficient in eliminating 70% or more of the bacterial load on inanimate high-touch surfaces, thus allowing short time for room turnover and potentially reducing pathogen transmission to patients and possibly SSI rates.

## Abbreviations

c.f.u.: Colony-forming units
IHP: Improved hydrogen peroxide products
IQR: Interquartile range
max: Maximum
min: Minimum
OR: Operating rooms
PX-UV: Pulsed xenon lamps
SSIs: Surgical site infections
TNTC: Too numerous to count

## References

[1] Shepard J, Ward W, Milstone A, Carlson T, Frederick J, Hadhazy E (2013). Financial impact of surgical site infections on hospitals; the hospital management perspective. JAMA Surg.

[2] Zimlichman E, Henderson D, Tamir O, Franz C, Song P, Yamin CK (2013). Health care–associated infections; a meta-analysis of costs and financial impact on the US health care system. JAMA Intern Med.

[3] Kramer A, Schwebke I, Kampf G (2006). How long do nosocomial pathogens persist on inanimate surfaces? A systematic review. BMC Infect Dis.

[4] Munoz-Price LS, Birnbach DJ, Lubarsky DA, Arheart KL, Fajardo-Aquino Y, Rosalsky M (2012). Decreasing operating room environmental pathogen contamination through improved cleaning practice. Infect Control Hosp Epidemiol.

[5] Yavuz SS, Bicer Y, Yapici N, Kalaca S, Aydin OO, Camur G (2006). Analysis of risk factors for Sternal surgical site infection emphasizing the appropriate ventilation of the operating theaters. Infect Control Hosp Epidemiol.

[6] Blanchard J (2009). Terminal cleaning. AORN J.

[7] Chemaly RF, Simmons S, Dale C, Ghantoji SS, Rodriguez M, Gubb J (2014). The role of the healthcare environment in the spread of multidrug-resistant organisms: update on current best practices for containment. Ther Adv Infect Dis.

[8] Simmons SE, Stachowiak J, Stibich M, Croteau M (2013). Using pulsed xenon ultraviolet to decrease contamination in operating rooms during terminal cleaning. Am J Infect Control.

[9] Fridman A, Bruno-Murtha LA, Osgood R, McAllister J (2013). Decreasing operating room contamination of surfaces and air with pulsed xenon ultraviolet disinfection. Am J Infect Control.

[10] Ghantoji SS, Stibich M, Stachowiak J, Cantu S, Adachi JA, Raad II (2015). Non-inferiority of pulsed xenon ultraviolet light versus bleach versus for reducing environmental *Clostridium difficile* contamination on high-touch surfaces in *Clostridium difficile* isolation rooms. J Med Microbiol.

[11] Stibich M, Stachowiak J, Tanner B, Berkheiser M, Moore L, Raad I (2011). Evaluation of a pulsed-xenon ultraviolet room disinfection device for impact on hospital operations and microbial reduction. Infect Control Hosp Epidemiol.

[12] Jinadatha C, Quezada R, Huber TW, Williams JB, Zeber JE, Copeland LA (2014). Evaluation of a pulsed-xenon ultraviolet room disinfection device for impact on contamination levels of methicillin-resistant *Staphylococcus aureus*. BMC Infect Dis.

[13] Stibich M, Stachowiak J (2016). The microbiological impact of pulsed xenon ultraviolet disinfection on resistant bacteria, bacterial spore and fungi and viruses. South. Afr J Infect Dis.

[14] Wiemken TL, Curran DR, Kelley RR, Pacholski EB, Carrico RM, Peyrani P (2014). Evaluation of the effectiveness of improved hydrogen peroxide in the operating room. Am J Infect Control.

[15] Havill NL, Havill HL, Mangione E, Dumigan DG, Boyce JM (2011). Cleanliness of portable medical equipment disinfected by nursing staff. Am J Infect Control.

[16] Qureshi Z, Mohamed MH (2013). Role of ultraviolet (UV) disinfection in infection control and environmental cleaning. Infect Disord Drug Targets.

